# Calf care workers' attitudes and personality and their association with calf mortality in large-scale dairy farms

**DOI:** 10.3389/fvets.2022.959548

**Published:** 2022-10-14

**Authors:** Dagni-Alice Viidu, Eamonn Ferguson, Tanel Kaart, Kerli Mõtus

**Affiliations:** ^1^Institute of Veterinary Medicine and Animal Sciences, Estonian University of Life Sciences, Tartu, Estonia; ^2^School of Psychology, University of Nottingham, Nottingham, United Kingdom

**Keywords:** dairy calf, on-farm mortality, calf care workers, perceptions, personality traits, mindset

## Abstract

Although calf mortality is a multifactorial problem, little is known about the attitudes and personalities of calf care workers (CCWs) and their association with calf mortality. This study aimed to describe the attitudes, satisfaction, and personality of CCWs in large Estonian dairy herds and to analyze their associations with herd calf mortality. A questionnaire registering CCWs' attitudes toward their work and calf mortality, personality characteristics, satisfaction and importance of different job-related factors was developed. In total, completed questionnaire data of 161 CCWs from 108 large (>100 cows) Estonian dairy farms were analyzed. Herd-level yearly calf mortality risk (MR) was calculated. Cluster analysis and variance partitioning analysis were applied to reveal the explanatory capacity of CCWs' attitudes and personalities on calf mortality. The mean yearly herd-level calf MR was 5.4% during the first 21 days of life and 2.7% during 22–90 days of life. Although good calf health and low calf mortality was important for CCWs, dead calves were often seen as inevitable. CCWs were generally doubtful regarding their capacity and available knowledge to influence calf mortality. In high-mortality herds, CCWs were dissatisfied with the calf health situation and farm working equipment and felt that the situation was out of their control. Despite striving, they had less faith that farmworkers could affect the outcomes, such as calf mortality. CCWs' personality domains explained <5% of the variance in the herd's calf MR, whereas their attitudes and satisfaction explained 20% of the variability in calf MR. The current study revealed the importance of the attitudes and satisfaction of CCWs on calf mortality and highlighted the need to allocate proficient assistance to herds with high calf mortality to mitigate calf health problems and the resulting consequences for CCWs.

## Introduction

Youngstock is the future of every dairy farm. Management decisions made during the pre-weaning period can affect the efficiency of the farm both directly *via* the number of available replacement heifers or sellable animals and indirectly by influencing calf health and productivity as lactating cows and the speed of the genetic progress of the herd (1, 2). During the pre-weaning period, calves are most vulnerable to diseases, and this period is characterized by the highest mortality (3–5). The calf mortality risks reported previously have differed widely, ranging between 1.5 and 13% during the first month of life (2, 6, 7), 0.6% and 9% during the first three months (5, 6, 8), and 1.3 and 6% during the first six months of life (7, 9, 10). Numerous studies have analyzed the reasons and risk factors for dairy calf mortality (11–13); however, increasing trends in calf on-farm mortality have been identified regardless of the excessive knowledge dissemination (7, 14).

Dairy herds have undergone extensive structural changes in recent decades, both in Estonia and elsewhere, during which the number of dairy herds has decreased while the average herd size has increased (15, 16). In 2020, 88% of Estonian dairy cow population was reared in herds with at least 100 cows, and since then, Estonian cows have also been the highest yielding cows in Europe (15, 17). With growing farm size, the structure of the farm has also become more sophisticated, and among other differences, the person in contact with the animals in such large commercial herds is mostly an employee rather than the farm owner (18).

According to the latest Estonian study including herds with >20 dairy cows, the predominant type of housing for calves <2 weeks old is individual pens in insulated barns, after which calves are mostly introduced to group pens; and during the first month of life, calves are mostly hand-fed milk twice a day (19). This results in high frequency of human-calf contact and indicates the paramount importance of calf care workers (CCWs) who provide daily care for calves during the preweaning period. In Estonia, CCWs generally have a large variety of responsibilities on the farm ranging across monitoring and assisting with calving, milking fresh cows and feeding calves, preparing and administering milk replacer, ensuring good pen hygiene, detecting diseased calves, and providing primary supportive health care. Attitudes are a predictor for human behavior (20) and farmers with more positive attitudes and gentle behavior regarding animals have been associated with better calf productivity and lower on-farm mortality (12, 13, 21). However, farm employees' perceptions, attitudes, and motivation might be entirely different from those of farm owners' and little is known on this topic. Regrettably, relatively few studies have focused on revealing the attitudes, personality and motivation of CCWs and analyzing them in the context of calf rearing outcomes, including on-farm mortality, and none of them have focused solely on large commercial farms (12, 22, 23).

There is also limited knowledge about the importance of and satisfaction of CCWs with different job-related factors. According to Herzberg's dual-factor theory, improving workers' satisfaction with hygiene factors is the basis for eradicating job dissatisfaction (24) and creating an opportunity to enhance workers' motivation. Well-motivated employees tend to be more productive, committed, and oriented toward meeting an enterprise's goals (24). According to Santman-Berends, et al. (13), having background information about farmers' mindsets could result in lower on-farm mortality *via* improved communication between veterinary advisors and farmers. In large farms, the dairy advisors interact with either directly the CCWs or, as more often happens, with the farm owner or the manager who later have to distribute the information within their team and ensure it is understood and accepted by all team members. Therefore, understanding the perceptions and values of CCWs and their motivation is a prerequisite for improving communication between dairy advisors and CCWs. In addition to economic and animal health benefits, lowering calf mortality would meet the expectations of the general public, as issues of animal welfare and sustainable production in modern dairy farms are the main concerns for consumers and individuals outside of the dairy industry (25).

The current study aimed to explore Estonian CCWs' attitudes, opinions, and satisfaction regarding calf mortality and to analyze the associations between their perceptions and personality traits and calf mortality. The study also aimed to reveal CCWs' satisfaction with, and the importance of, several factors related to working conditions and -environment.

## Materials and methods

### Herd recruitment

A list of herds with at least 100 cow-years (cow-years were computed by dividing the sum of annual number of feeding days of the entire cow herd by the number of days per year) at the beginning of 2019 was obtained from Estonian Livestock Performance Recording Ltd. and included 182 herds. In this study, a herd was defined as a dairy unit(s) of cows managed as one operation together with any associated youngstock unit(s). Considering the available resources and size of the study population, we targeted 120 herds for inclusion in the study. Additional inclusion criteria were a loose-housed keeping system for milking cows and no intention to cease production in the near future. A random sample of 120 farms was obtained from the list of herds using a random number generator in Stata^®^ MP14.2 (College Station, TX: StataCorp LP). Farms were contacted individually through telephone to ascertain compliance with the inclusion criteria and explain the objectives and methodology of the study. If a contacted herd did not meet the inclusion criteria, a new herd was randomly selected from the sampling frame. A total of 169 herds were contacted before a final sample of 120 herds was obtained.

### Questionnaire

Each questionnaire began with a cover letter specifying the aim and scope of the study, information about the funding, participants' confidentiality, optional nature of participation, and contact details of the principal investigator. The cover letter attached to the questionnaire also specified that the participants give their consent to participate in the study by filling in and returning the questionnaire. The theoretical framework of the questionnaire was based on previous research regarding dairy farmers and focused on specific empathy and attitudes toward animals (26, 27), personality (28), general empathy (29), job satisfaction (30), quality of life (27, 31), perceived control over their job (12), and sociodemographic profile (21). The first part of the survey included questions about the respondents (age, sex, level of education, number of years of working experience on the current farm and with cattle in total, and the age of the calves they work with), followed by statements from eight themes: “importance of mortality” (number of statements *n* = 5), “satisfaction with mortality” (*n* = 1), “responsibility regarding calf mortality” (*n* = 2), “attitude and empathy toward calves” (*n* = 4), “self-confidence” (*n* = 5), “job satisfaction and motivation” (*n* = 2), “quality of life” (*n* = 4), and “empathy” (*n* = 4). Questions on satisfaction with different job-related factors (number of factors offered = 9) and the importance of different job-related factors (*n* = 14) were also included. The exact statements are listed in Table 1. Finally, the Ten-Item Personality Inventory (TIPI) was used to assess CCWs' personality characteristics (28).

**Table 1:** Statements reflecting calf care workers' attitudes and opinions, personality characteristics and satisfaction with work-related factors evaluated in a 7-point Likert scale and Spearman correlation coefficients with calf mortality in two age groups.

**Block / variable name**	**Statement**	**Mean ±SD (min, max)** ***n = 161^a^***	**Correlation with yearly calf mortality risk**	
			**0–21 days old calves n = 157^**a**^**	**22–90 days old calves** ***n = 150^a^***
**Importance of mortality**				
b1_imp_low_mort	It is important for me that not many calves die	6.9 ± 0.6 (1, 7)	−0.03	−0.15*
b1_mort_welfare	If a lot of calves die their welfare is poor	5.1 ± 2.0 (1, 7)	−0.04	−0.08
b1_mort_workload	If a lot of calves die my workload is higher	4.6 ± 2.3 (1, 7)	0.14*	0.14*
b1_mort_resolv_own	Calf mortality is a problem that will resolve by itself	1.6 ± 1.3 (1, 7)	−0.01	0.19*
b1_mort_inevit	Having dead calves is inevitable	3.6 ± 1.9 (1, 7)	−0.06	0.00
**Satisfaction with mortality**				
b2_sat_mort	I am satisfied with the calf mortality level on our farm	3.8 ± 2.2 (1, 7)	−0.36*	−0.39*
**Responsibility regarding calf mortality**				
b3_mort_treat	High calf mortality is a result of poor veterinary care	2.7 ± 1.7 (1, 7)	0.03	−0.08
b3_mort_staff	Calf health issues and mortality are affected by people working with calves	4.5 ± 2.2 (1, 7)	−0.05	−0.12*
**Attitude and empathy toward calves**				
b4_dead_calves_sad	Seeing a dead calf makes me sad	6.5 ± 1.2 (1, 7)	−0.06	0.04
b4_calves_pain	Calves feel physical pain just as humans do	6.8 ± 0.6 (4, 7)	0.06	0.15*
b4_sick_calves_sad	Sick calves make me sad	6.7 ± 0.8 (3, 7)	−0.03	0.06
b4_drink_effort	If the calf is not drinking, I will make an effort to ensure it drinks	6.6 ± 1.0 (1, 7)	0.15*	0.14*
**Self-confidence**				
b5_know_low_mort	I know exactly what needs to be done to have low calf mortality	4.4 ± 1.7 (1, 7)	0.03	0.07
b5_good_at_job	I am good at my job	5.4 ± 1.4 (1, 7)	0.04	−0.04
b5_skills_know	I have sufficient knowledge and skills to do my job well	5.5 ± 1.4 (1, 7)	0.00	0.09
b5_prob_indep	In case of calf-related problems I can find a solution on my own	4.5 ± 1.6 (1, 7)	−0.03	−0.05
b5_overcome_calf_prob	Calf health problems are not under my control	4.0 ± 1.7 (1, 7)	0.18*	0.06
**Job satisfaction and motivation**				
b6_like_job	I like my job	6.5 ± 0.9 (4, 7)	−0.06	−0.11*
b6_mot_job_well	I am motivated to do my job well	6.1 ± 1.4 (1, 7)	−0.02	−0.03
**Quality of life**				
b7_qual_life_imp	Raising the quality of my life is important to me	6.3 ± 1.3 (1, 7)	0.06	0.01
b7_health_imp	My health is more important for me than work	5.0 ± 1.8 (1, 7)	0.03	0.00
b7_hobbies	It is important for me to have hobbies outside of work	5.1 ± 2.1 (1, 7)	−0.01	−0.04
b7_fam_friends	It is important for me to spend time with friends and family	6.0 ± 1.6 (1, 7)	−0.04	−0.10*
**Empathy**				
b8_upset_affects	If someone is upset it affects me too	5.1 ± 2.2 (1, 7)	0.02	0.09
b8_help_emot	I know how to help others deal with bad emotions	4.3 ± 2.1 (1, 7)	−0.15*	−0.04
b8_share_emot	I often share my emotions with others	3.9 ± 2.4 (1, 7)	0.08	−0.03
b8_critiz_situat	Before I criticize others, I try to put myself into their situation	4.9 ± 2.1 (1, 7)	−0.01	0.08
**Big-Five personality domains**				
big5_extraversion	Extraversion	3.7 ± 1.3 (1, 7)	0.11*	−0.09
big5_agreeableness	Agreeableness	5.8 ± 1.1 (2.5, 7)	0.01	0.04
big5_conscientiousness	Conscientiousness	6.3 ± 0.9 (2.5, 7)	−0.03	−0.04
big5_emotional_stability	Emotional stability	5.2 ± 1.4 (2.0, 7)	0.04	−0.01
big5_openness_to_experiences	Openness to new experiences	5.5 ± 1.2 (2.5, 7)	0.00	−0.05
**Satisfaction with work-related factors**				
In my current workplace I am satisfied with…				
g1_sat_salary	…size of the salary	4.4 ± 1.7 (1, 7)	−0.04	−0.01
g1_sat_equipment	…working equipment	5.1 ± 1.6 (1, 7)	−0.14*	−0.20*
g1_sat_environment	…working environment	5.1 ± 1.7 (1, 7)	−0.09	−0.09
g1_sat_workload	…amount of work assignments	4.9 ± 1.8 (1, 7)	−0.06	−0.04
g1_sat_schedule	…working schedule	5.9 ± 1.5 (1, 7)	−0.01	−0.08
g1_sat_boss_att	…supervisors' attitude toward my job	5.5 ± 1.6 (1, 7)	−0.02	−0.04
g1_sat_self_improv	…self-education opportunities	4.4 ± 2.1 (1, 7)	−0.03	0.03
g1_sat_feedback	…getting feedback about my work	5.0 ± 1.9 (1, 7)	−0.04	−0.10*
g1_sat_coll_workqual	…quality of co-workers' work	4.8 ± 1.6 (1, 7)	0.06	0.02

a Number of calf care workers' responses.

* p-value < 0.25.

A 7-point Likert scales were used to record responses. For statements in blocks 1–7 and for TIPI, these were from “completely disagree” (= 1) to “completely agree” (= 7). For the statements in the theme “empathy”, the extremities were named “does not describe me well” for score 1 to “describes me very well” for score 7. For the questions regarding satisfaction with different job-related factors, these were from “not satisfied at all” to “very satisfied” and for the questions about importance of different job-related factors as motivators, they were “not important at all” and “very important” for scores 1 and 7, respectively. The middle answer (score 4) was named as “so and so”. The questionnaires were composed in both Estonian and Russian and were pre-tested by the CCWs of three farms to verify the understandability of the questions and response scale. Approval for this study was obtained from the University of Tartu Ethics Committee for Human Research (Protocol no. 292/T-18, date 15.04.2019).

One to four anonymous pre-developed questionnaires were sent to each study farm to be filled out by CCWs who had been working in the farm with pre-weaned calves for at least the last 12 months. The number of questionnaires sent to each farm was specified during the telephonic conversation when contacting the farms and introducing them to the study. A prestamped envelope was included with each questionnaire, and the completed copies were returned either by post or directly to the researcher during the farm visit to guarantee the anonymity of the respondent.

### Data collection and statistical analysis

According to Estonian legislation, calves must be ear-tagged within the first 20 days of life (32); therefore, the registry data might miss some calf mortality cases during the first three weeks of age. To calculate the yearly calf mortality risk (hereafter also “calf on-farm mortality” or “calf mortality”) during the first 21 days of life [hereafter: younger age group (YAG)], data on the number of live births and occurred deaths (including unassisted death and euthanasia) were gathered from the farm records. Similar data for calves aged 22–90 days [hereafter: older age group (OAG)] were obtained from the Estonian Agricultural Registers and Information Board. Calf mortality risk for both age groups were calculated for each individual herd for the period of 1 year preceding the farm visit [Equations (1) and (2)]. All statistical analyses were performed separately for the two age groups, and only the questionnaires of the CCWs who worked with the respective calf age group were included in the analysis.


$$\begin{array}{lll} yearly\;calf\;mortality\;risk\;\left(YAG\right)\; & = & \frac{yearly\;number\;of\;calves\;died\;before\;22\;days\;of\;age}{yearly\;number\;of\;live-born\;calves\;}\;\times \;100\;\left(1\right)\\ yearly\;calf\;mortality\;risk\;\left(OAG\right)\; & = & \frac{yearly\;number\;of\;calves\;died\;between\;22-90\;days\;of\;age}{yearly\;number\;of\;calves\;present\;in\;the\;farm\;at\;22-90\;days\;of\;age}\;\times \;100\;\left(2\right) \end{array}$$

All farms were visited once between August 2019 and July 2020, and all questionnaires were obtained by August 2020. The questionnaires were digitalized with an electronic survey tool LimeSurvey (LimeSurvey GmbH), exported to an Excel spreadsheet, and combined with the data regarding the number of births, deaths, and mortality risk. Data analysis was performed using R software version 3.6.1 (33).

To avoid losing herds or questions due to missing values, multiple imputation of the data was performed using the random forest algorithm with the R package missForest (34). Correlations between the studied statements (continuous or ordinal data) and yearly calf mortality risk were examined using Spearman correlation analysis. The statistical associations between categorical variables (sex, education level) and calf mortality risk were examined using analysis of variance (ANOVA). To avoid omitting variables with a relatively weak association, but potentially useful in common patterns analysis, a liberal *p*-value of <0.25 was used (35) as the selection criterion to determine the variables to be used in later analyses. To generate CCWs' subgroups based on their attitudes and personality traits, a k-means clustering algorithm was applied to the preselected variables (marked with an asterisk in Table 1). The R package NbClust (36) was used to determine the optimal number of clusters and to perform the analysis. Tukey *post-hoc* test and Kruskal-Wallis test, respectively, were used for comparisons of the mortality risks and preselected variables to detect statistically significant difference in mean values between the clusters at *p* ≤0.05. Finally, variance partitioning analysis (VPA) was performed using the R package vegan (37) to analyze the relative importance of the four groups of variables (“Respondent”, “Farm”, “Attitudes and satisfaction”, and “Big5”) on calf mortality. The group “Respondent” included data about the respondents' sex, age, education level, and working experience with cattle in total and on the current farm. The group “Farm” contained information about the number of calves born on the farm within 1-year period preceding the farm visit (YAG) or the number of calves in the age group of 22–90 days of age present on the farm during the one-year period preceding the farm visit (OAG). The group “Attitudes and satisfaction” included the same preselected variables that were used in the cluster analysis, and the group “Big5” consisted of data from the Ten-Item Personality Inventory, which was converted according to the method developed by Gosling, et al. (28) to form five personality domains (extraversion, agreeableness, conscientiousness, emotional stability, and openness to experiences).

## Results

### Farm and respondent characteristics

**Figure 1:**
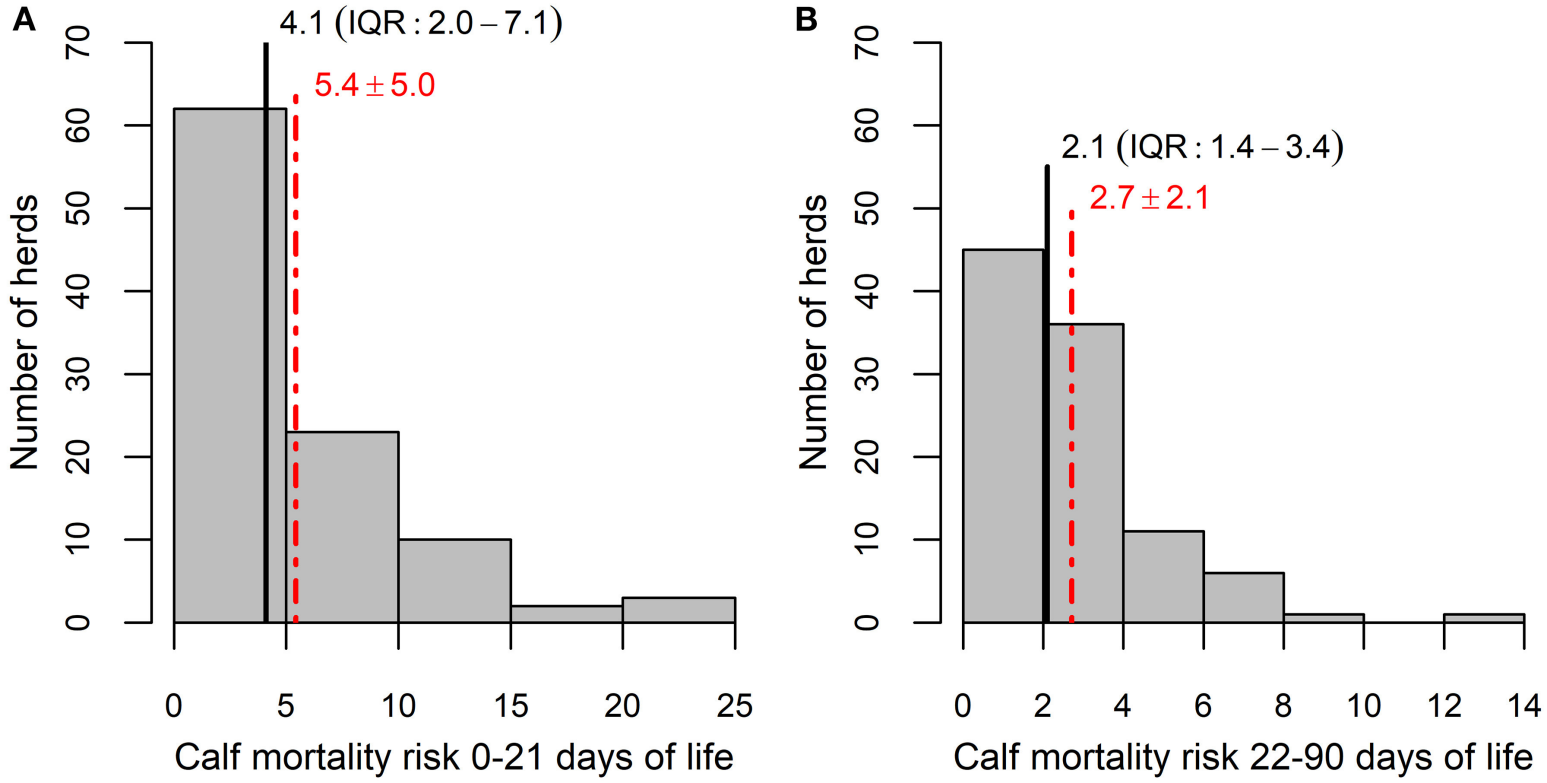
Distribution of herds according to yearly calf mortality risk during the first 21 days of age **(A)** and 22–90 days of age **(B)**. The red dotted line shows the mean value and red numbers represent the mean ± standard deviation, black line shows median and black numbers represent the median and interquartile range (IQR).

The median herd-level calf mortality risk was 4.1% (mean 5.4%, range 0.0–23.3%) during the first 21 days of age and 2.1% (mean 2.7%, range 0.0–12.7%) during 22–90 days of age (Figure 1). Herd-level calf mortality risk of calves up to 21 days of age was positively correlated with the herd-level mortality risk of 22–90 days old calves (*r* = 0.49, *p* < 0.001).

Because some farms did not have any CCWs who wished or were eligible to respond to the questionnaire, CCWs from 108 farms were included in the study. A total of 176 questionnaires were received, of which 153 were in Estonian and 23 in Russian. 15 questionnaires were excluded from the analyses as the respondent had <1 year of working experience on the current farm, thus having less potential impact on the yearly calf mortality risk. Of the 161 respondents included in the analysis, 146 (90.7%) worked with calves from both age groups, while 15 worked with only one of the two age groups (11 with YAG and four with OAG); therefore, the number of questionnaires used in the analyses was 157 and 150 for YAG and OAG, respectively. In total, there were 82 missing answers (0.78%) among 161 respondents and 65 questions. The maximum number of missing answers per question was five, while 22 questions (33.9%) were fully answered. Forty-two questionnaires (26.1%) had at least one missing answer, with a maximum of 11 missing answers. Of 161 respondents, 17 (10.6%) were men and 144 (89.4%) were women. The average working experience with cattle was 20 years and mean age of the CCWs was 47.1 and 47.5 years in the YAG and OAG, respectively (Table 2). The majority of the respondents had general or upper secondary vocational education (*n* = 86, 53.4%), followed by basic (*n* = 42, 26.1%), higher or professional higher (*n* = 16, 9.9%), vocational (*n* = 15, 9.3%), and primary (*n* = 2, 1.2%) education. At a *p*-value limit of 0.25, Spearman correlation analysis or analysis of variance reported no statistically significant associations between calf mortality and general questions regarding the respondent in the YAG; however, in the OAG, female sex was associated with lower mortality risk (*p* < 0.01). The numerical variables describing the farms and respondents are presented in Table 2.

**Table 2:** Descriptive statistics of farm and respondent variables and their Spearman correlation coefficients with yearly calf mortality risk.

**Variable**	**Mean** ±**SD (min, max)**		**Correlation with yearly calf mortality risk**	
	**0–21 day old calves'** **keepers (*****n*** = **157)**	**22–90 day old calves'** **keepers (*****n*** = **150)**	**0–21 day old calves**	**22–90 day old calves**
Number of calves	553.8 ± 366.0 (90, 2007)^a^	423.1 ± 266.5 (69, 1358)^b^	−0.04	−0.04
Age of the respondent (years)	47.1 ± 12.1 (18, 73)	47.5 ± 12.0 (21, 73)	0.01	−0.04
Working experience in the current farm (years)	10.7 ± 9.2 (1, 41)	10.6 ± 9.1 (1, 41)	0.01	0.09
Working experience with cattle in total (years)	19.6 ± 13.0 (1, 65)	19.8 ± 13.1 (1, 65)	−0.06	0.02

a Number of calves born during one year before the farm visit.

b Number of calves of the respective age group present in the farm within one year preceding to the farm visit.

### Calf care workers' attitudes toward calves, calf mortality, and their work

CCWs found it to be important that not many calves die [mean score (MS) ± SD = 6.9 ± 0.6] and, on average, were neutral regarding the satisfaction with the calf mortality level on their farm (MS = 3.8 ± 2.2). Respondents tended to agree that if many calves die their workload is higher (MS = 4.6 ± 2.3) and that calf mortality is also an indication of poor calf welfare (MS = 5.1 ± 2.0). On average, respondents neither agreed nor disagreed that calf mortality is inevitable (MS = 3.6 ± 1.9); however, they disagreed that the calf mortality problem would resolve by itself (MS = 1.6 ± 1.3). CCWs rather disagreed that high calf mortality is a result of poor veterinary care (MS = 2.7 ± 1.7) and were generally unsure whether calf health issues and mortality are affected by the people working with calves (MS = 4.5 ± 2.2). On average, CCWs were uncertain if they have sufficient knowledge to achieve low calf mortality (MS = 4.4 ± 1.7) and were hesitant in their ability to overcome calf-related problems on their own (MS = 4.5 ± 1.6). They were also doubtful to claim that the calf health situation was under their control (MS = 4.0 ± 1.7).

**Figure 2:**
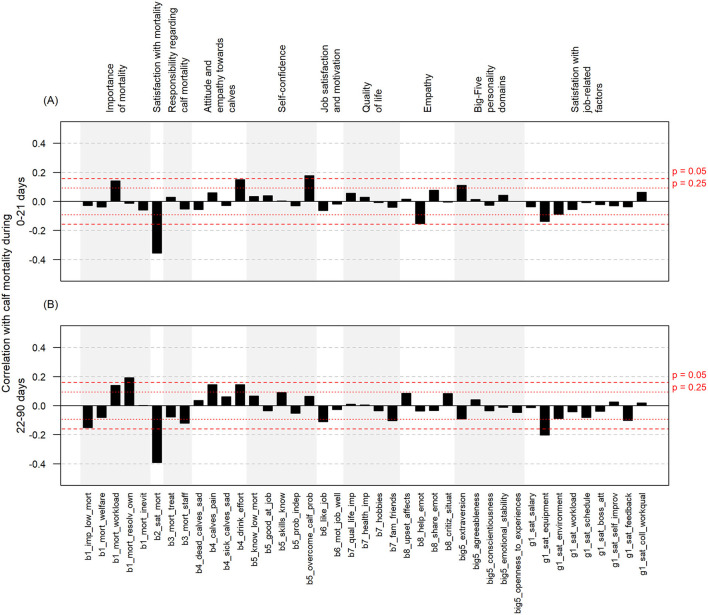
Spearman correlations between calf care workers' attitudes, opinions, personality domains, and satisfaction with job-related factors and yearly calf mortality risk (%) during the first 21 days of age **(A)** and 22–90 days of age **(B)**. Abbreviations are explained in Table 1.

The mean, minimum, and maximum scores of all the statements regarding the attitudes, personality, and satisfaction of the respondents are presented in Table 1, along with the correlation with calf mortality in both age groups. The distribution of CCWs' answers to specific statements and correlations with calf on-farm mortality are also presented in Supplementary Figure 1 and Figure 2, respectively.

### Calf care workers' clusters and association with calf on-farm mortality

**Figure 3:**
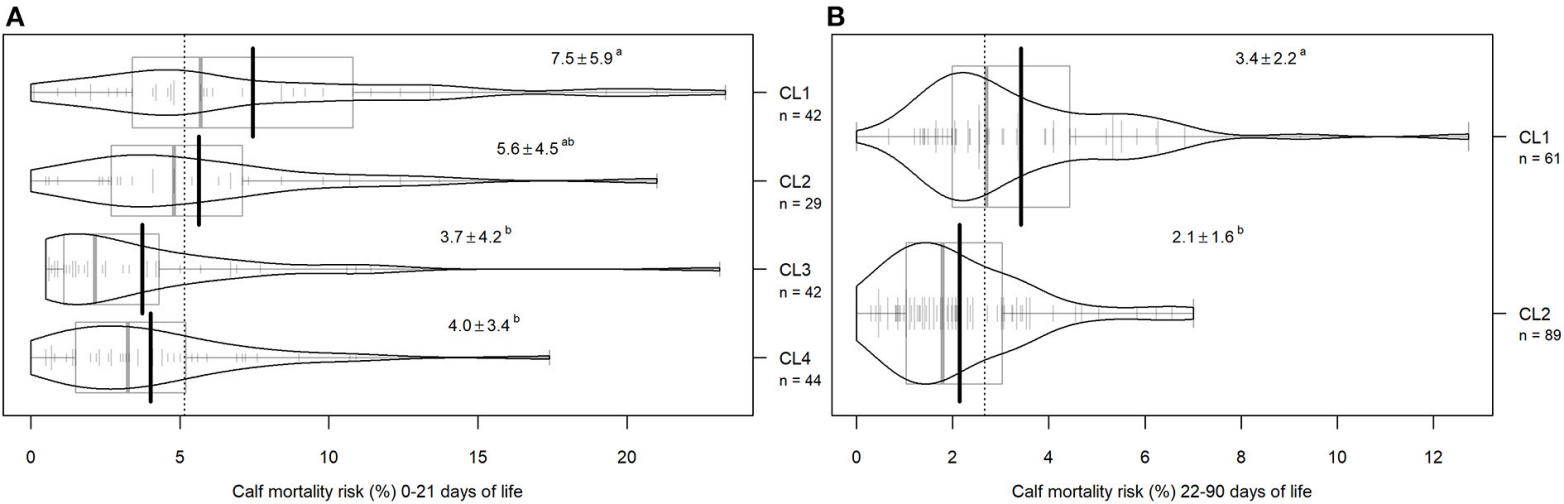
Distribution of yearly calf mortality risk during the first 21 days of age **(A)** and 22–90 days of age **(B)** in each cluster (CL) formed in k-mean cluster analysis. Small vertical lines indicate calf mortality risk of a single respondent's farm, bold vertical gray lines show cluster-based median, and bold black vertical lines denote cluster-based means. Gray squares illustrate the interquartile range and the dotted line marks the overall mean. Mean ± standard deviation is shown numerically for each cluster and different superscript letters indicate statistically significantly different clusters according to the Tukey *post-hoc* test.

**Figure 4:**
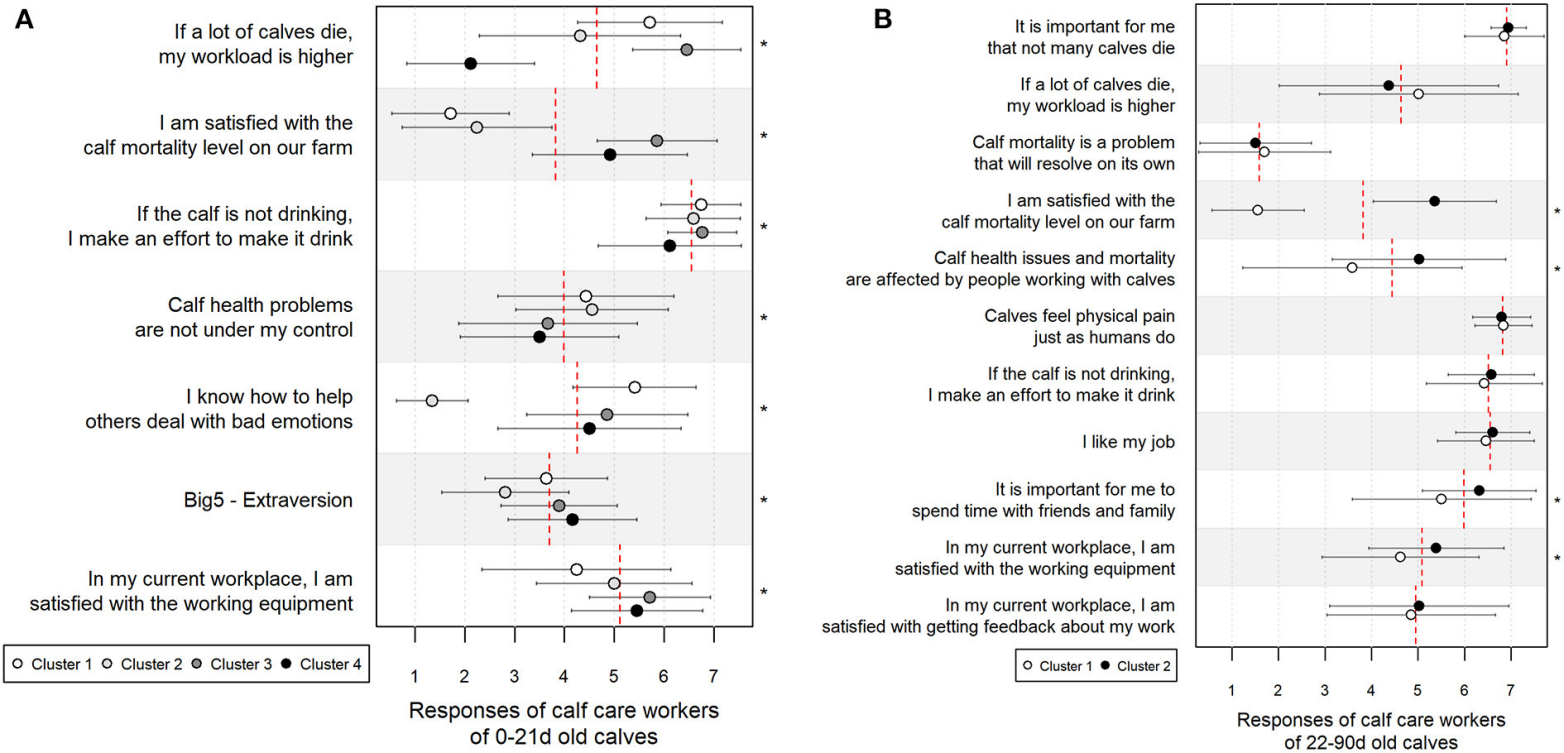
Mean ± standard deviation of the responses (1 = complete disagreement, 7 = strong agreement) of the selected variables across the calf care workers' subgroups created in the k-mean cluster analysis. During the first 21 days of age **(A)** the mean ± standard deviation of the calf mortality risk in the cluster (CL) is: CL1 = 7.5 ± 5.9, CL2 = 5.6 ± 4.5, CL3 = 3.7 ± 4.2, CL4 = 4.0 ± 3.4. For calves of 22–90 days of age **(B)**, the mean ± standard deviation of mortality risk is CL1 = 3.4 ± 2.2 and CL2 = 2.1 ± 1.6. The variables are selected based on their correlation with yearly calf mortality risk (see Table 1 for correlations). The red dotted lines mark the overall means and the stars (*) on the right side of the plot indicate a statistically significant difference between the clusters in Kruskal–Wallis test at *p* < 0.05.

Seven variables with a *p*-value <0.25 in correlation analysis were eligible for the cluster analysis in the YAG and 11 in the OAG (Table 1). Cluster analysis produced four clusters in the YAG [number of respondents in cluster (CL) 1 = 42, CL2 = 29, CL3 = 42, and CL4 = 44] and two clusters in the OAG (number of respondents in CL1 = 61 and CL2 = 89). The mean calf mortality risk across the clusters is shown in Figure 3. A significant difference in mortality risk was identified between CL1 and CL3 and between CL1 and CL4 (*p* < 0.01) in the YAG and between the two OAG clusters (*p* < 0.001). The mean scores per cluster for the statements used in the cluster analysis are presented in Figure 4.

In the YAG analysis, we found that in the cluster of herds with significantly higher yearly calf mortality risk (CL1), CCWs were less satisfied with the calf mortality level in their farm (CL1 vs. CL3 and CL1 vs. CL4, *p* < 0.001), thought that higher calf mortality increases their workload (CL1 vs. CL4, *p* < 0.001), were more likely to make an effort to ensure the calf drinks in case it does not do so willingly (CL1 vs. CL4, *p* = 0.03), and were less satisfied with the working equipment in the farm (CL1 vs. CL3, *p* < 0.001 and CL1 vs. CL4, *p* = 0.002). CCWs from CL1 herds also felt more often that they know how to help other people deal with negative emotions (CL1 vs. CL4, *p* = 0.02). No significant association was identified in the cluster analysis between the extraversion of CCWs from CL1 compared to CL3 and CL4 (Figure 4, panel A).

In the analysis of OAG, it appeared that in the cluster of herds with significantly higher yearly calf mortality risk (CL1), CCWs were less satisfied with the calf mortality level (*p* < 0.001) and the working equipment on the farm (*p* = 0.003), perceived it to be less important to spend time with friends and family (*p* = 0.002), and agreed less often that calf health issues and calf mortality are affected by the people working with calves (p < 0.001), compared to CCWs from CL2 (Figure 4, panel B).

### Importance of different factor groups on calf mortality

**Figure 5:**
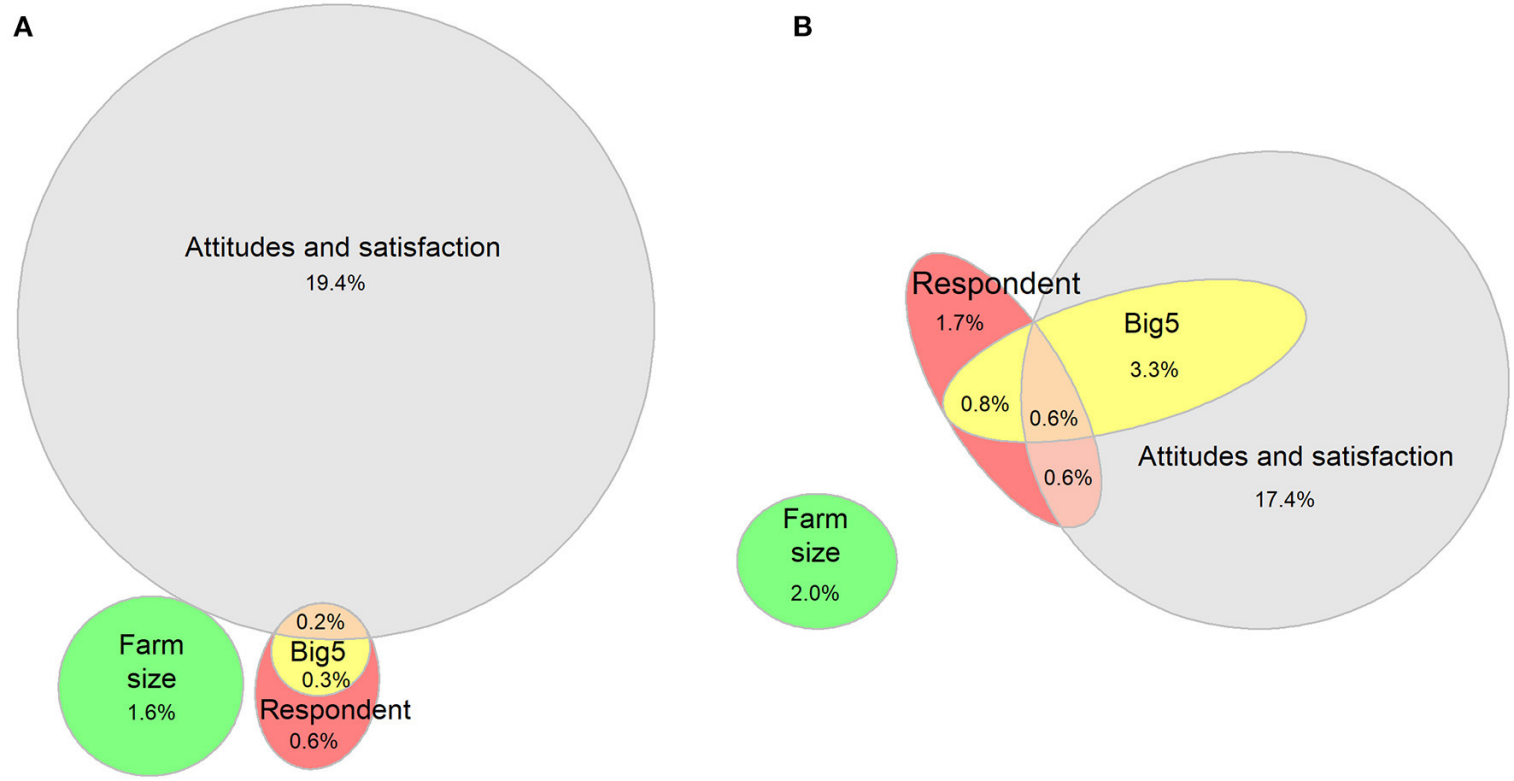
Euler diagram of the results of variance partitioning analysis for yearly calf mortality risk during the first 21 days **(A)** and 22–90 days of age **(B)**. The numerical values and size of the ellipses and their intersections visualize the variance (%) of yearly calf mortality risk described by farm size (number of calves); respondent-specific traits (age, sex, education level, and working experience); selected calf care workers' attitudes; opinions; and satisfaction (6 and 11 questions with *p* < 0.25 in correlation analysis with yearly calf mortality risk during the first 21 days of age and 22–90 days of age, respectively) and all Big Five personality domains.

VPA showed that the respondents' attitudes and satisfaction explained 19.6% of the total variability in the mortality risk of YAG (Figure 5), and most of the explained variance in calf mortality risk was attributed to this group of variables alone (R^2^ = 19.4%). The respondent-specific traits and Big Five personality domains had marginal explanatory capacities (R^2^ = 1.1% and R^2^ = 0.5%, respectively), with respondent traits alone describing only 0.6% of the variance in yearly calf mortality in this age group. The effect of personality domains was entirely covered by the other groups of variables.

According to the VPA, a total of 21.9% of the variance in yearly calf mortality risk was explained by respondents' attitudes and satisfaction in the OAG analysis, and these variables alone explained 17.4% of the variance. The respondent-specific traits accounted for 3.7% and the Big Five personality domains for 4.7% of the total variability in mortality in the OAG. The effect of the personality domains was completely included in the effect of other groups, whereas respondent traits alone accounted for 1.7% of the variability. The variance explained by farm size was approximately the same in both age groups (R^2^ = 1.6% and R^2^ = 2.0% in the YAG and OAG, respectively), and none of the effect was explained by any other group of variables.

### Calf care workers' satisfaction with and the importance of work-related factors

**Figure 6:**
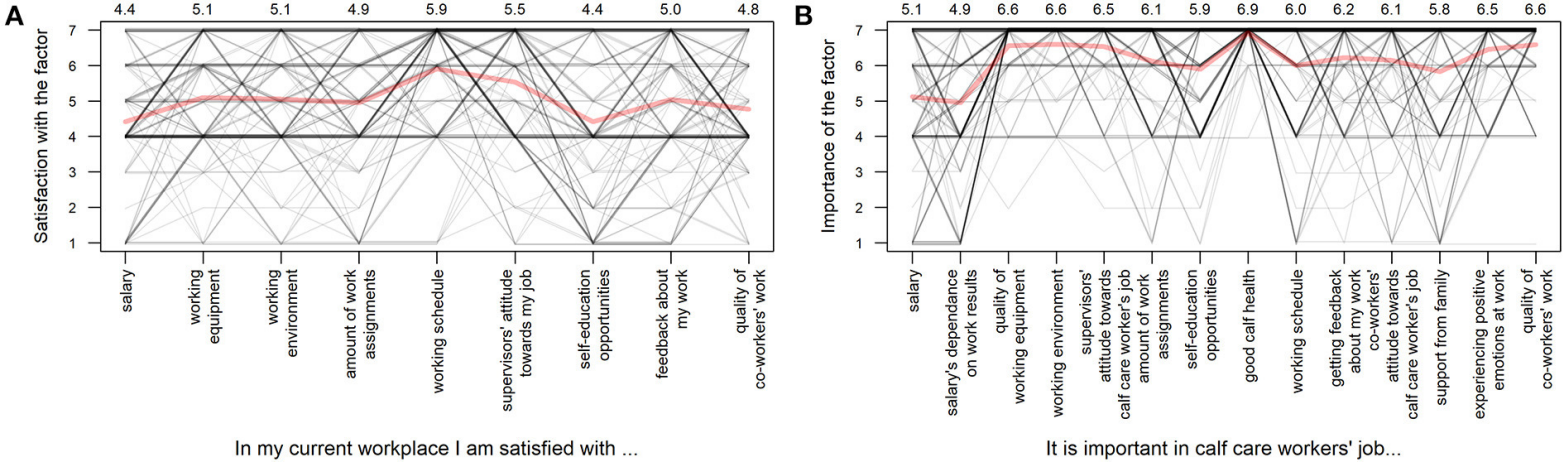
The average scores on a 7-point Likert scale (1—not satisfied/important at all, 7—very satisfied/important) for statements regarding the calf care workers' satisfaction with **(A)** and the importance of **(B)** different work-related factors (*n* = 161 responses). The gray lines denote the individual answers and the red line represents the average score which is also shown numerically on top of the figure.

The analysis of satisfaction with and the importance of different job-related factors revealed almost identical results for both age groups. The maximum divergence of the mean scores between the responses of the CCWs from the two age groups was 0.1 points and are thus presented jointly (Figure 6). Although satisfaction was moderate even with the lowest-scoring factors, out of all the proposed factors, CCWs were least satisfied with salary (MS = 4.4 ± 1.7) and self-education opportunities (MS = 4.4 ± 2.1) and most satisfied with working schedule (MS = 5.9 ± 1.5) and supervisors' attitude toward their job (MS = 5.5 ± 1.6). The most important factors in CCWs' work were stated to be good calf health (MS = 6.9 ± 0.4), working environment (MS = 6.6 ± 0.9), quality of working equipment (MS = 6.6 ± 0.9), and quality of co-workers' work (MS = 6.6 ± 1.0). The least important factors among those studied were salary (MS = 5.1 ± 2.0) and salary's dependence on work results (MS = 4.9 ± 2.0). The detailed results with the mean scores for each factor are presented in Figure 6.

## Discussion

### Calf on-farm mortality, farm and respondent characteristics

The mean calf mortality risks were 5.4% and 2.7% during the first 21 and 22–90 days of age, respectively. Previous studies have also found that mortality is higher in earlier life and have detected similar mortality values (2, 5, 9, 38), as well as substantially smaller or larger values (4, 6–8, 10, 14, 39). The year-long period chosen for calf mortality risk calculation in this study aids in minimizing the possible seasonal patterns of mortality (7, 11, 39) and makes the results more comparable. Nevertheless, different age category margins and calculation methods between studies still make it difficult to reasonably compare these results. No comprehensive report regarding the overall pre-weaned calf mortality risk in commercial farms, or specifically the mortality of calves up to 21 days of age, has been published in Estonia previously, making this the first publication to report these values and create a baseline for further comparisons.

The current study included only large commercial dairy herds that entailed a different complexity of factors compared to smaller herds. Investigating the impact of farm's environmental and management-related factors on calf mortality was beyond the scope of the present study, and only herd size, considered a proxy for many other farm factors (40), was included. A larger farm size, which has often been linked to increased calf mortality (2, 3, 19, 39), was not significantly associated with calf mortality risk in this study. As herd size was one of the criteria in the herd recruitment process (only herds with >100 cows were included), the study population of the current study was presumably more harmonized regarding the overall production conditions compared to some of the above-cited studies.

The people responsible for providing calf care in large-scale dairy herds are generally employees. The calf mortality rate is higher in farms where paid labor is used to manage calves (41–43), and little is known about their attitudes, work motivation and resulting behavior, and whether and how this relates to calf health. According to a review by Adler, et al. (26), personality is what defines a person, differentiates them from others, and remains largely unchanged after 30 years of age, whereas attitudes change depending on the context and are always determined by the object. The average calf care worker in our study farms was a 47 years old person with a long working experience, meaning that they probably have a well-developed personality, accompanied by distinguished beliefs and attitudes toward calves and their work in general and deeply rooted work routines. Changing their mindset and habits might be more challenging; however, farm- or worker-specific approaches have turned out to be effective in lowering calf mortality (13). Benchmarking has also been shown to be beneficial for justifying some changes in calf management (44). Except of the respondent's sex in the OAG analysis (female sex was associated with lower mortality), none of the general respondent characteristics was found to be associated with calf mortality. The detected association was too weak to make any generalized conclusions; however, women are considered to be more empathetic toward others than men and apply a more caring approach toward calves, which could elucidate this tendency (21, 27, 45).

A moderate positive correlation was detected between the mortality risks of the two age groups of calves. Although completely different pathogens and problems affect calves of different age bands during the first 90 days of life (46), we can speculate that common farm-level factors affect the health and welfare of calves of different age groups. As the overlap of staff working with both age groups of calves was relatively high, personnel attitudes and behavior might also affect calf mortality similarly in both age groups.

### Calf care workers' attitudes and opinions and association with calf mortality

CCWs generally had a positive attitude toward calves, as indicated by the high empathy toward calves and highly expressed importance of low calf mortality. Having a more positive attitude and behavior toward calves decreases stress and mortality and increases animal welfare and productivity, whereas negative beliefs have the opposite effect (21, 22, 27, 30, 47). CCWs generally enjoyed their work and felt motivated to perform well. Owing to their long average working experience, they are probably well-accommodated to work with calves.

CCWs perceived high calf mortality as a welfare issue and a problem that is not self-dissolving. They also tended to acknowledge the personal consequences accompanying high levels of calf mortality by mostly agreeing that this entails an increased workload. The latter statement was positively correlated with calf on-farm mortality, meaning that CCWs in high-mortality herds probably feel somewhat overwhelmed by the increased workload. Managing sick calves consumes more time, and the subsequent unpredictable time needed to accomplish the rest of the work assignments might deepen the feeling of losing control over the situation (12). However, people's perception of control over the situation determines their motivation to take action in the hope of solving the issue rather than categorizing it as unavoidable or inevitable (12). The feeling of losing control over calf health problems was somewhat more prevalent in high-mortality herds, but feeling powerless in the face of high mortality might make people more susceptible to implementing the necessary changes (13).

CCWs from herds with high calf mortality appeared to be slightly more self-focused, reflected by the lower importance of spending time with family or friends, and showed a tendency for less extraversion. This indicates a more reserved nature and possibly a higher level of loneliness, which might result in reduced occupational well-being (31, 48). Professional assistance should be allocated to herds with high on-farm calf mortality, and in addition to composing a systematic mitigation plan, providing physical and mental support is important to assist CCWs in overcoming the situation.

On average, regardless of herd calf mortality rates, CCWs were hesitant about whether calf mortality is inevitable. Even though high calf mortality, as a problem, was not entirely ignored, some level of adaptation to calf mortality was evident. To some extent, CCWs have become accustomed to the concept of calves dying on farms and probably consider it a reality that accompanies this profession. Mee (49) has concluded this mindset as, “*where there are livestock there are deadstock*.” Being accustomed to calf deaths might be due to both the overall and farm-based low prioritization of calf mortality. In Estonia, calf mortality data are not routinely summarized in any way, resulting in a lack of fundamental information for adequate assessment of the calf health situation in the farms. It also complicates any herd-based comparisons or benchmarking that could help bring more attention to the calf mortality issue (44). If farmers or farm managers do not see the value of lowering mortality, an unfavorable basis is created to implement any changes. In some countries, such as the Netherlands, calf mortality rates are calculated for all herds (14) and can be used for different monitoring purposes as well as to determine the need for more advisory services to herds with higher mortality. Mortality is also an indicator of herd-level welfare and can be used to assess the need for such improvements (10).

A positive attitude describing CCWs' commitment and efforts (“*if the calf is not drinking, I will make an effort to ensure it drinks*”) was identified, with somewhat higher scores in high-mortality herds. In response to witnessing distress and more mortality, the CCWs may become more compassionate and try harder to help the animal in need. At the same time, CCWs were mostly unsure whether staff working with calves have a substantial impact on calf health issues and the resulting mortality, especially in high-mortality herds. We can speculate whether CCWs thought their personal contribution and individual responsibility regarding the calf health situation on the farm was marginal or unrewarding despite efforts, or simply believed the effect of the human-animal relationship, in general, to be negligible in determining the overall calf health outcomes. Poor veterinary care was usually not blamed for calf deaths, and CCWs generally perceived their knowledge and skills to be sufficient to perform their duties. Simultaneously, their self-confidence in handling calf health problems efficiently and finding solutions on their own was rather mediocre. We can assume that CCWs might see other limiting factors beyond their and co-workers' control, such as disease-causing infections or farm conditions, being responsible for calf mortality. Due to the design of the study, we are unsure about the cause-effect direction in this case—whether CCWs' low self-confidence affects calf mortality or whether high calf mortality impairs their self-belief despite of striving. In addition, it remains to be confirmed whether CCWs have a self-justification attitude and distance themselves from the problems in herds with high calf mortality.

Although we do not know the direction of causality between the studied statements and herd's calf mortality level, it became obvious from this study that “farm blindness” was not prevalent among the CCWs in our herds. This was evidenced by the significant correlation between CCWs' lower satisfaction with the calf mortality level on their farms and the real increased herd calf mortality. According to Mee (50), “farm blindness” is a misperception where people who work on a farm, day-to-day, falsely think that what they observe every day on their own farm is normal and similar to what occurs in every other farm, when in reality it is not. This misleading thinking prevents the problem from being addressed, as it is not really perceived as a problem. In large-scale Estonian dairy herds, CCWs clearly perceive the existence of a problem; however, they also feel powerless to make a substantial difference. Nevertheless, feeling powerless over the situation can also be classified as one stage of awareness (13), and acknowledgment is the first step toward unraveling the issue.

### Importance of different factor groups on calf mortality

In this study, the attitudes and satisfaction of CCWs explained approximately one-fifth of the variability in calf mortality, whereas CCWs' personality traits had negligible explanatory capacity. Previous studies have also found that rather than personality, the attitudes of workers influence the outcome of farming (20, 27). The respondent-specific characteristics, which had a rather marginal descriptive capability altogether, had a larger overlap with personality domains than with attitudes. This is probably due to personality remaining relatively unchanged after early adulthood (26). Age is also reflected in the education level and working experience of the person, while the attitudes are context-dependent. In the employee recruitment process, most attention is generally paid to persons' education and previous working experience; however, as these factors appear to play a lesser role in achieving the desired calf rearing outcomes, perhaps more emphasis should be placed on finding ways to assess people's attitudes toward calf rearing and calf health issues upon employment. In contrast to personality, attitudes can also predict human behavior (20) and this presumably expresses in the ways the workers communicate with and take care of the animals. Addressing calves with care and compassion results in less reactivity and stress, higher calf welfare, and lower mortality (21, 22, 47).

Herd size appeared to have a distinct but overall limited explanatory capacity for calf mortality. Contrary to farm managers of the same study population (51), herd size did not overlap with the attitudes and opinions of CCWs, referring that their perceptions and attitudes were unaffected by the overall farm factors. As the study population was rather homogeneous and comprised of only large commercial farms, more farm-level factors would have to be included in the analysis to draw any generalized conclusions about their role in herd calf mortality level.

### Calf care workers' satisfaction with and the importance of work-related factors

According to Herzberg's two-factor theory, different intrinsic and extrinsic factors affect people's job satisfaction (24). In this study, CCWs were least satisfied with self-education opportunities and salary and were most satisfied with working schedule and supervisors' attitude toward their job, although the level of satisfaction was modest overall. All but the first are considered extrinsic or hygiene factors in Herzberg's theory and are directed toward eradicating job dissatisfaction, while self-education opportunities are considered intrinsic or motivating factors (24). Because of the generally low level of self-confidence in combating calf health problems and dissatisfaction with self-education possibilities, we can assume that CCWs might feel that they are alone and armless in facing calf health issues; thus, further activities supporting their knowledge and ability to cope should be promoted.

Although CCWs did not show an acceptable level of satisfaction with their salary, they interestingly did not consider it to be a highly important factor in their job either. Eliminating job dissatisfaction is nevertheless a precondition for increasing the motivation of workers; therefore, farm owners should focus on ensuring the expected wage levels. Working equipment is another key component, and the moderate satisfaction score given by CCWs shows plenty of room for advancement. Due to the existing association between lower satisfaction with working equipment and increased calf mortality, improvements in the implements could potentially lead to better calf health.

The focus of employee motivation should be on improving calf health on the farm, possibly by incorporating veterinary or herd health advisors for an effective mitigation plan. In addition, while material aspects are understandably a fundamental part of every employment relationship, many farm owners and managers may underestimate employees' interest in self-education (52). Offering more frequent, interesting, or engaging training opportunities can enhance employees' motivation and increase their efforts to meet farm goals. As farm owners and employees might have different understandings of the farm's goals (52), these should be discussed and agreed upon by the farm staff. The more vigorous involvement of CCWs in farm planning and goal setting probably also increases their feeling of being an essential part of the team. Being recognized, along with having good working conditions, increases job satisfaction as well (24, 31).

### Study limitations

The current study was a part of a larger project which aimed to analyze different farm-level factors and their associations with cow and calf mortality. In smaller herds (<100 cows) calves are usually managed by the farmer itself or other family members, while in larger farms paid labor is used. Considering this conceptual difference and the ongoing intensification of dairy farming, we aimed to include only CCWs of large commercial farms and to have a rather unified study population, only loose housed farms were included.

As Estonian farmers must ear-tag their calves during the first 20 days of life (32), the most reliable data about on-farm calf mortality could be obtained from the farm records. It can be argued whether farm workers (both in one specific farm as well as between the study farms) always use the same threshold levels for discriminating between stillbirth and mortality of live-born calves during registration. As was shown by Santman-Berends, et al. (14) even slight differences in the definitions of parameters can have a major effect on the obtained calf mortality values. Some level of inaccuracy can therefore exist across the study farms in the mortality risk of YAG; however, we do not think that this registration issue could bias the study results as we believe it to be independent of the herd calf mortality risk.

As the majority of Estonian CCWs are women, the gender distribution of the respondents in our study was uneven. However, as our results indicate that CCWs' sex itself is not a strong predictor for calf mortality (while the attitudes and satisfaction are), our results can be extrapolated to all Estonian CCWs taking into account their unbalanced gender distribution.

We also could not control for the individual contribution of each CCWs. Although we asked which age group of calves the CCWs work with and included their answers only to the respective age group analyses, calf management systems differ greatly across the farms. For example, on one farm the CCW might hand-feed the calves for 5 days after which the calves are fed with an automatic feeder while on another farm hand-feeding period might be 14 or 21 days. As CCWs have different kind of responsibilities across the farms, we also do not know how much time the respondents actually spent working with calves in the respective age group.

Lastly, due to the historical single cohort study design we cannot make causal inferences for the identified associations.

## Conclusions

The present study confirmed that CCWs acknowledge the importance of good calf health; however, at some level, calf mortality was seen as an inevitable reality of farming. CCWs generally felt insecure regarding combating calf mortality problems and were unsure about the relevance of their role in achieving low mortality. They understood the negative consequences of high calf mortality, for example, increased workload and greater efforts needed from them, suggesting that in herds with high calf mortality, their work motivation was probably also affected. Therefore, we suggest that improved visibility and higher prioritization of calf mortality are preconditions for improvements. Creating a support system allocating professional consultations in herds with high calf mortality could lead to expected calf health outcomes.

The current study revealed that CCWs' satisfaction with the calf mortality level of the farm was negatively correlated with calf mortality risk, indicating that “farm blindness” is rarely the case among CCWs on commercial Estonian farms. Although CCWs' personality traits had negligible explanatory capacity for herd calf mortality level, the study results confirmed the relevance and substantial impact of CCWs' attitudes and opinions on calf health outcomes with approximately 20% of the variance in calf mortality being explained solely by the intrinsic factors of the person working with calves. Therefore, special training of dairy advisors and farm managers is needed to be able to consider the different perceptions and attitudes of CCWs in an attempt to improve the overall communication and farming outcome.

According to the CCWs' expressed satisfaction, there is room for improvement regarding most of the studied work-related factors. Low satisfaction with working equipment and receiving less feedback about their work was also associated with higher calf mortality. Therefore, improving working conditions is a preliminary step in removing the dissatisfaction of CCWs and possibly promoting calf health. Ensuring good calf health was the most important factor for CCWs in their work, and improving this could lead to better work motivation. The results of this study supplement the current knowledge on calf mortality problems and offer valuable input for developing a more comprehensive approach to mitigating calf mortality.

## Data availability statement

The datasets presented in this article are not readily available because according to the confidentiality information provided to the participants upon giving their consent to participate in this study, only the principal investigator and collaborators directly involved in the study will have access to the completed questionnaire data, and forwarding the data to third parties is not permitted. Requests to access the datasets should be directed to D-AV, dagni-alice.viidu@emu.ee.

## Ethics statement

The studies involving human participants were reviewed and approved by University of Tartu Ethics Committee for Human Research. The patients/participants provided their written informed consent to participate in this study.

## Author contributions

D-AV, KM, and EF contributed to conception and design of the study. D-AV organized the database. D-AV and TK performed the statistical analysis. D-AV and KM wrote the first draft of the manuscript. All authors contributed to manuscript revision, read, and approved the submitted version.

## Funding

This study was supported by an Estonian Research Council Grant (PSG268). The funding body had no role in the design of the study, data collection, data analysis, interpretation of results, or writing of the manuscript.

## Conflict of interest

The authors declare that the research was conducted in the absence of any commercial or financial relationships that could be construed as a potential conflict of interest.

## Publisher's note

All claims expressed in this article are solely those of the authors and do not necessarily represent those of their affiliated organizations, or those of the publisher, the editors and the reviewers. Any product that may be evaluated in this article, or claim that may be made by its manufacturer, is not guaranteed or endorsed by the publisher.

## Abbreviations

CCW: calf care worker
CL: cluster
MR: mortality risk
MS: mean score
OAG, older age group: calves of 22–90 days of age
TIPI: Ten-Item Personality Inventory
VPA: variance partitioning analysis
YAG, younger age group: calves up to 21 days of age.
