# Supplementary figures and images for: Calf care workers' attitudes and personality and their association with calf mortality in large-scale dairy farms
Source: Front Vet Sci. 2022 Oct 14;9:959548. doi: 10.3389/fvets.2022.959548 (PMC9614149; doi:10.3389/fvets.2022.959548)

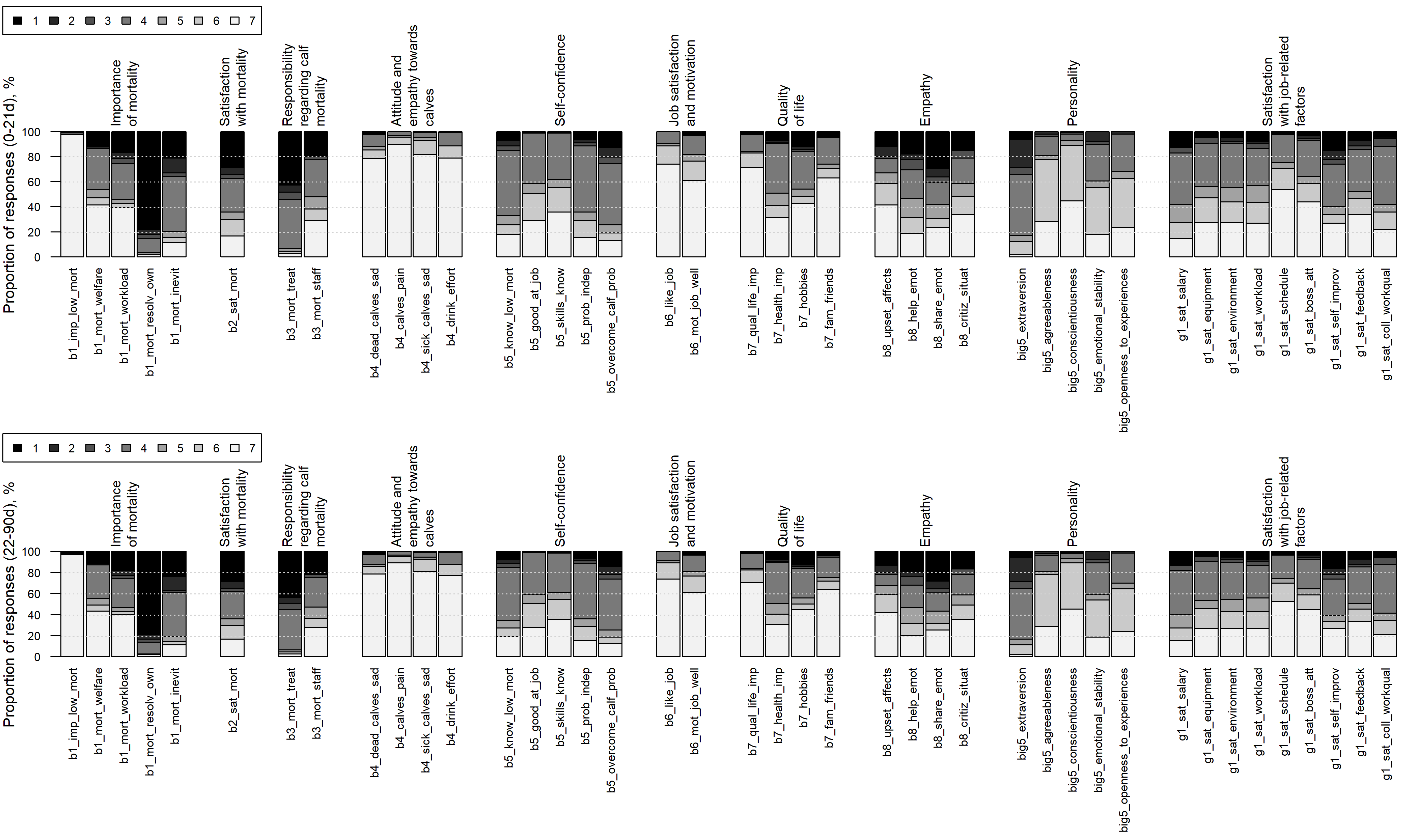

Supplement: Supplementary file 1 [file Image_1.TIFF]
